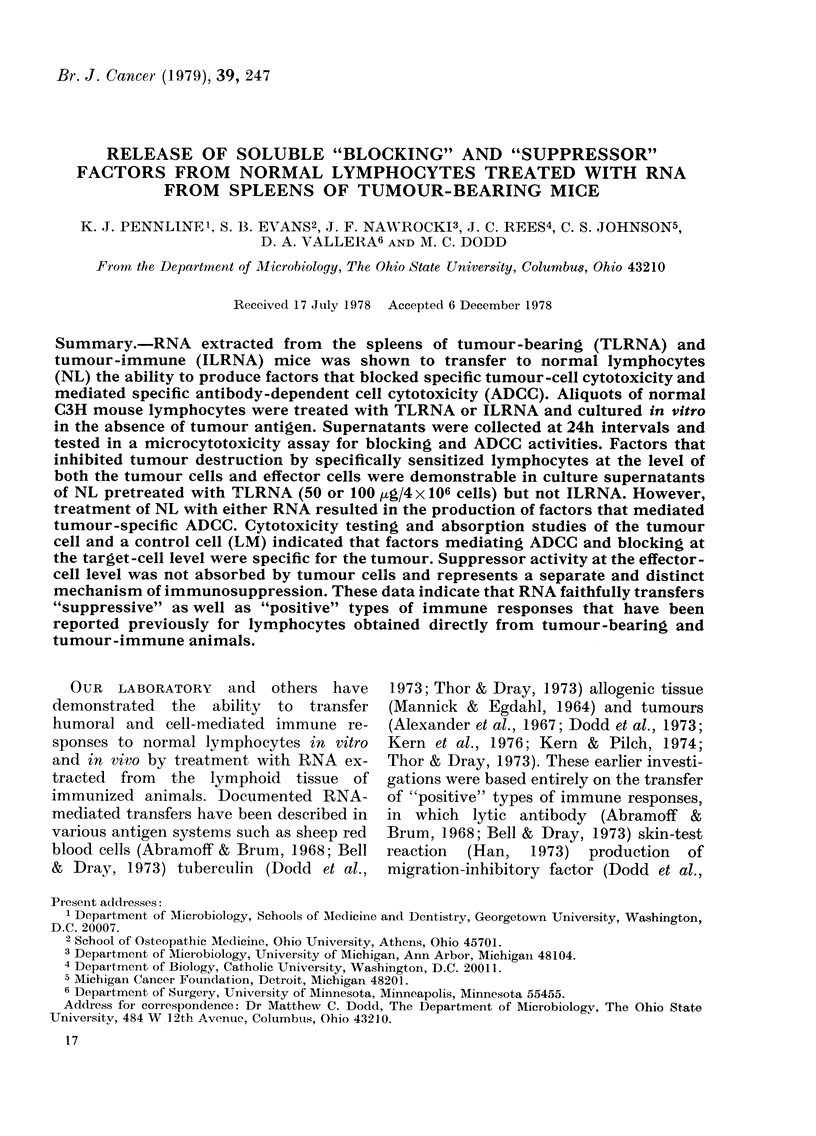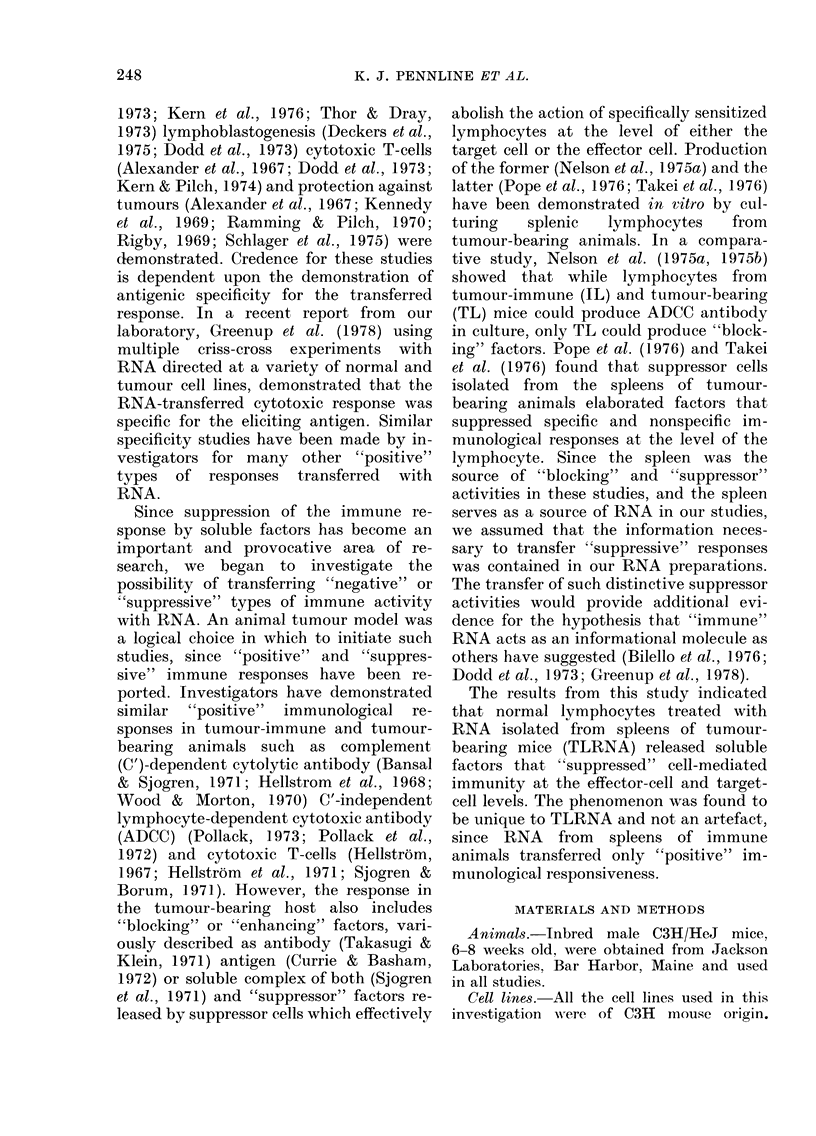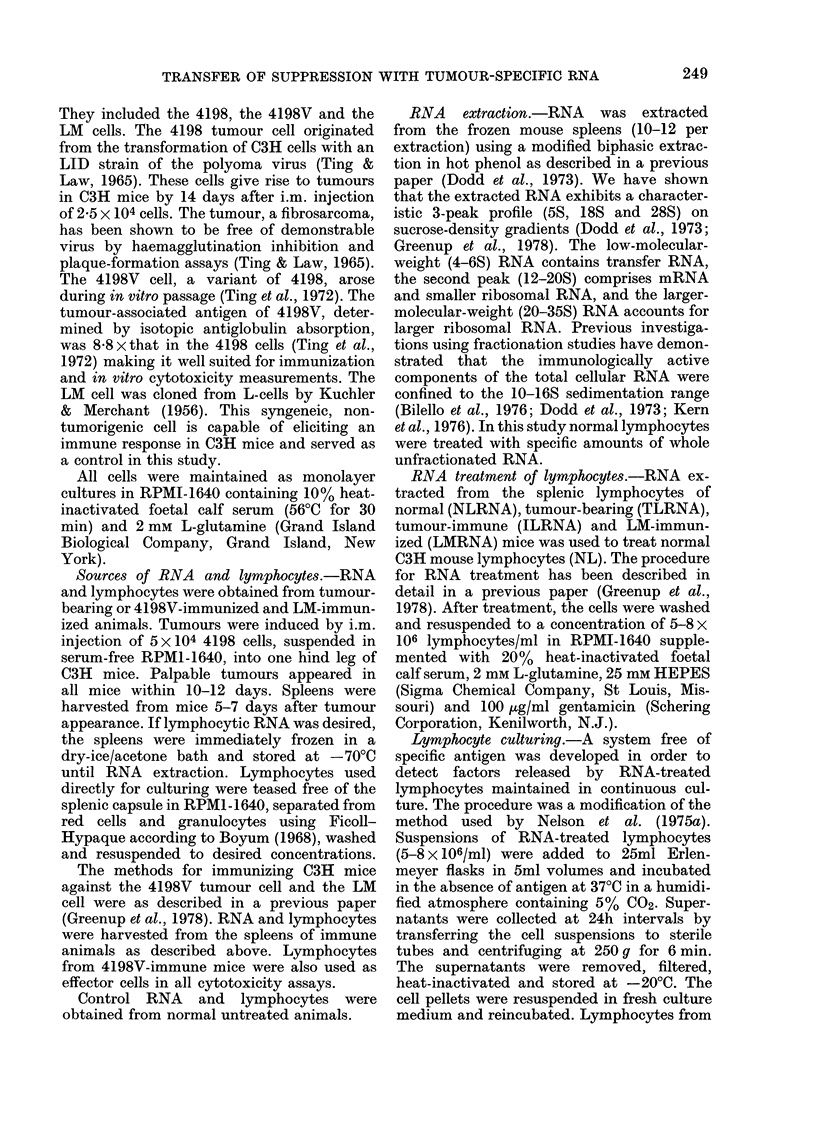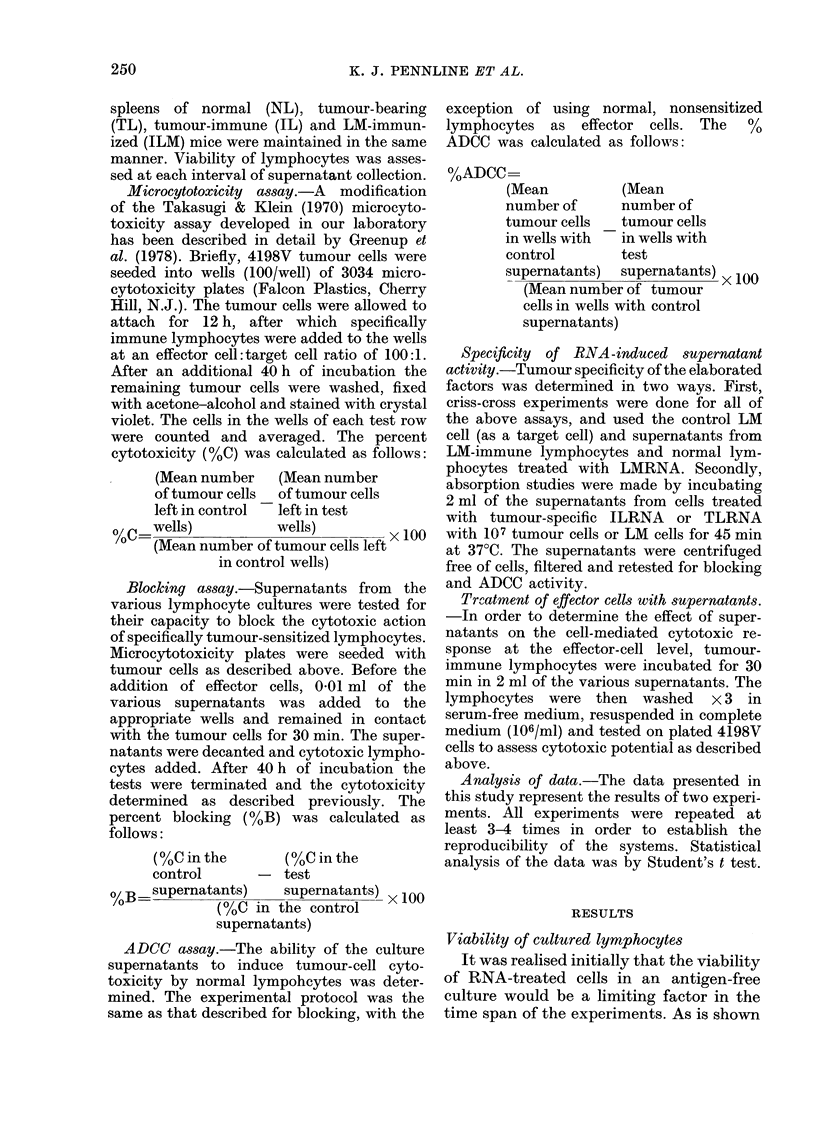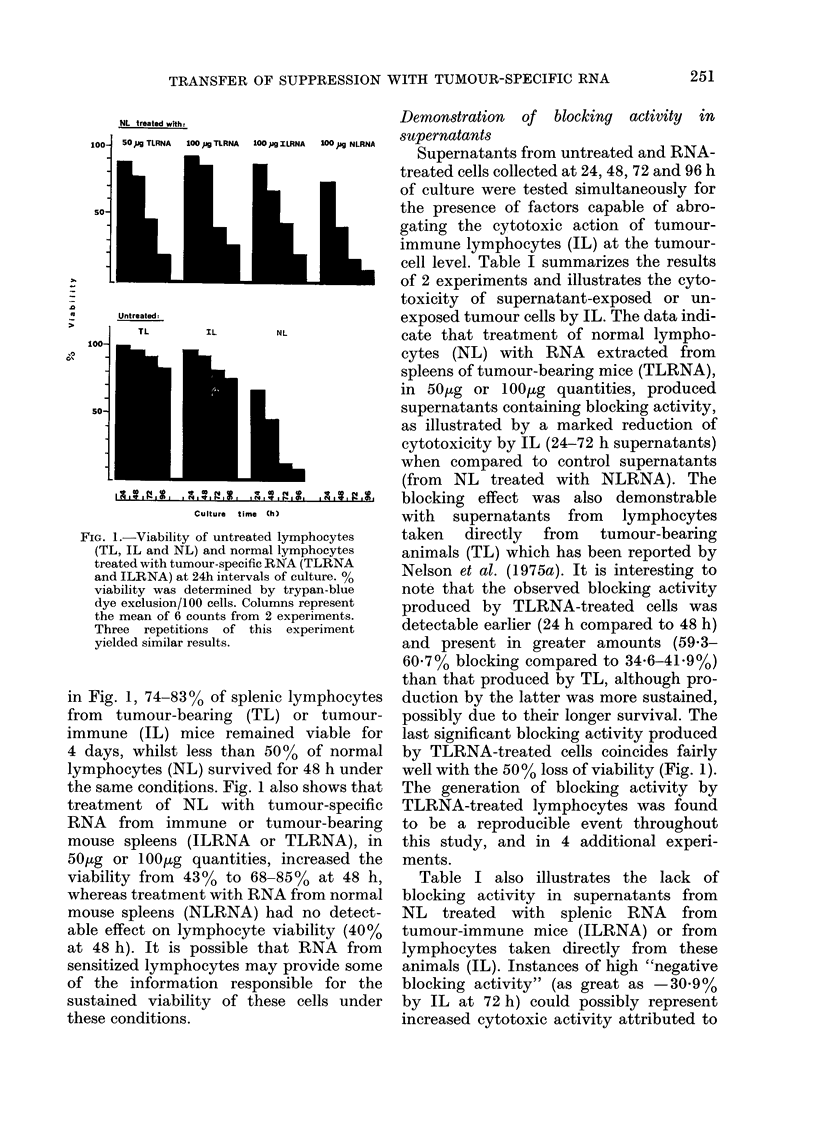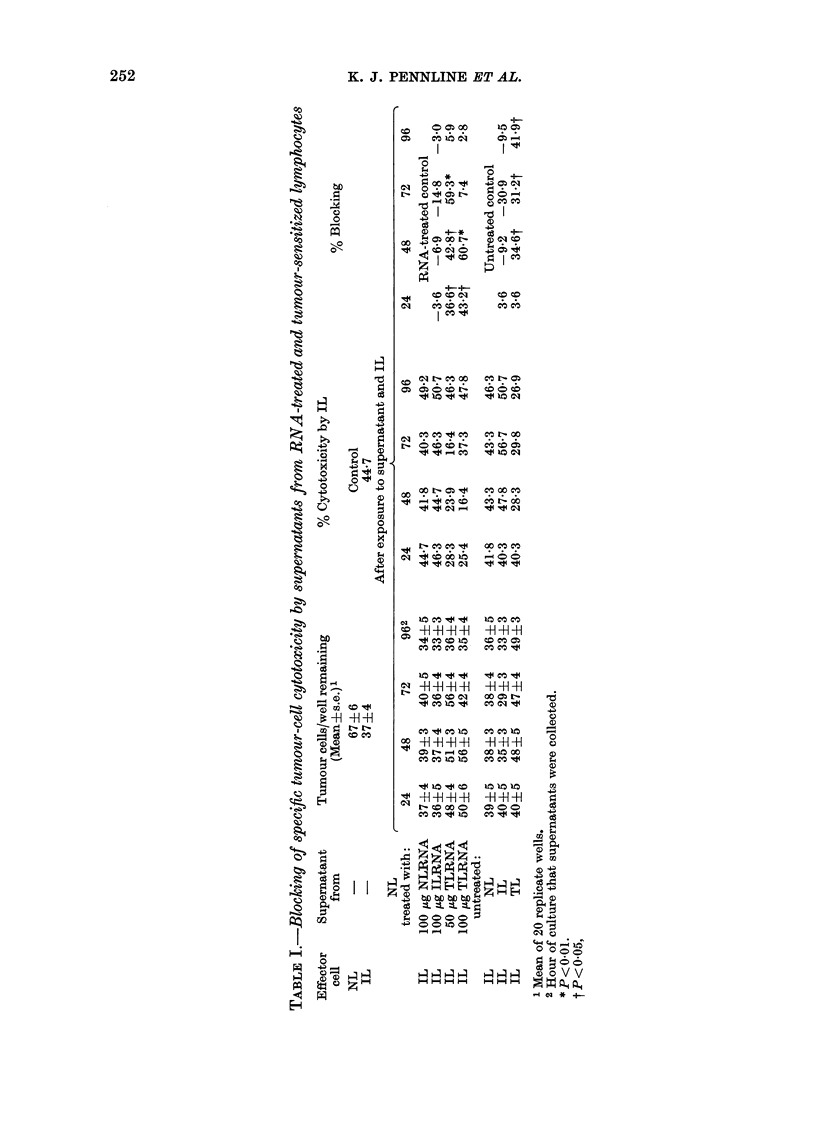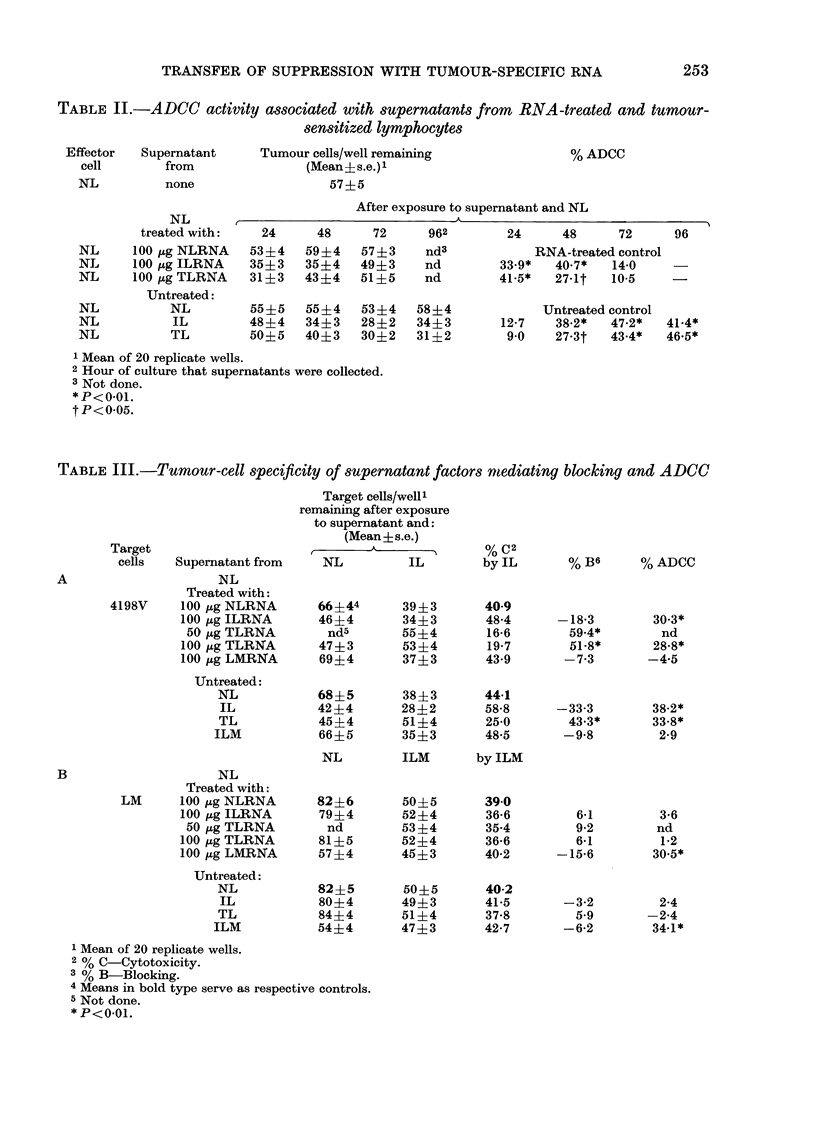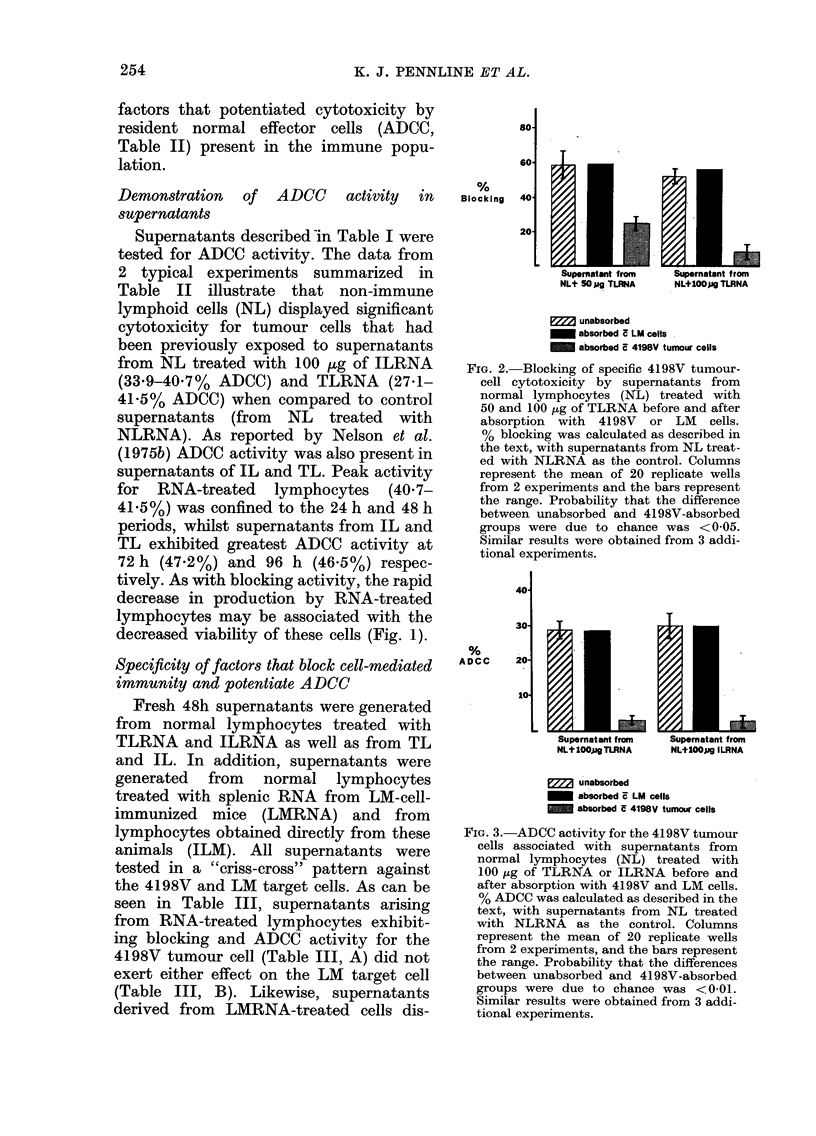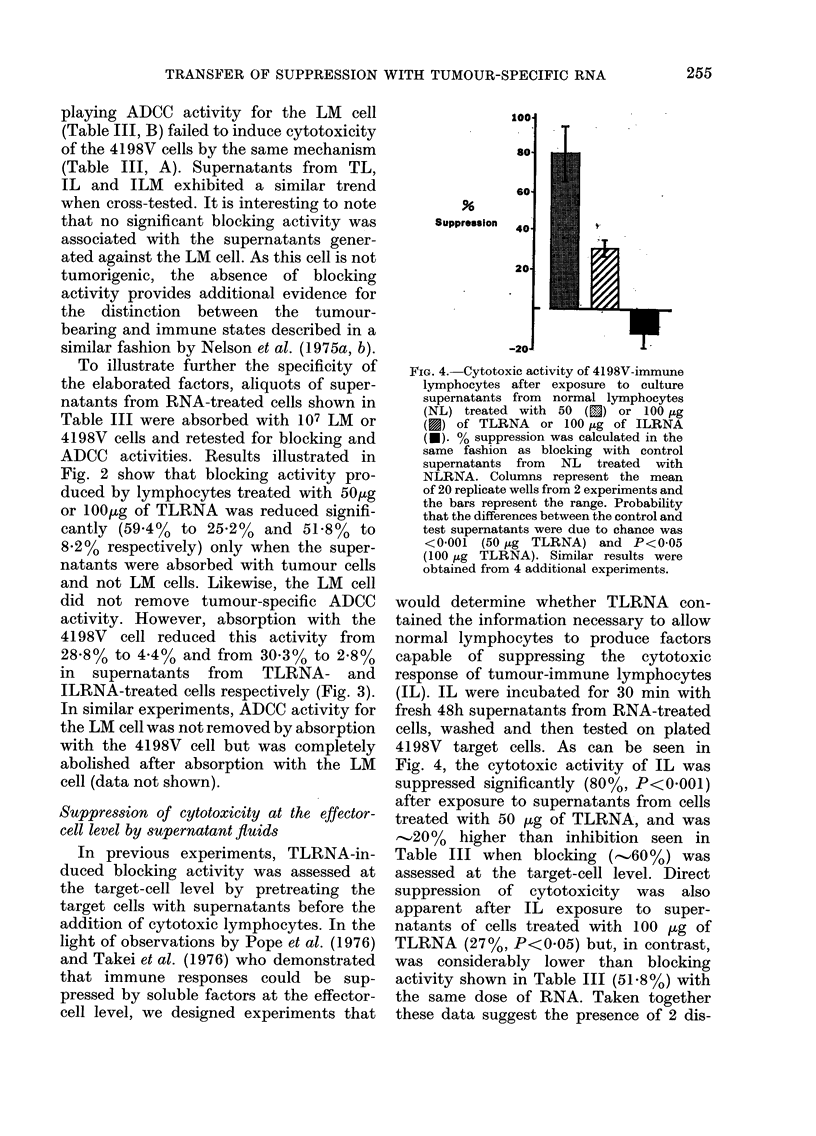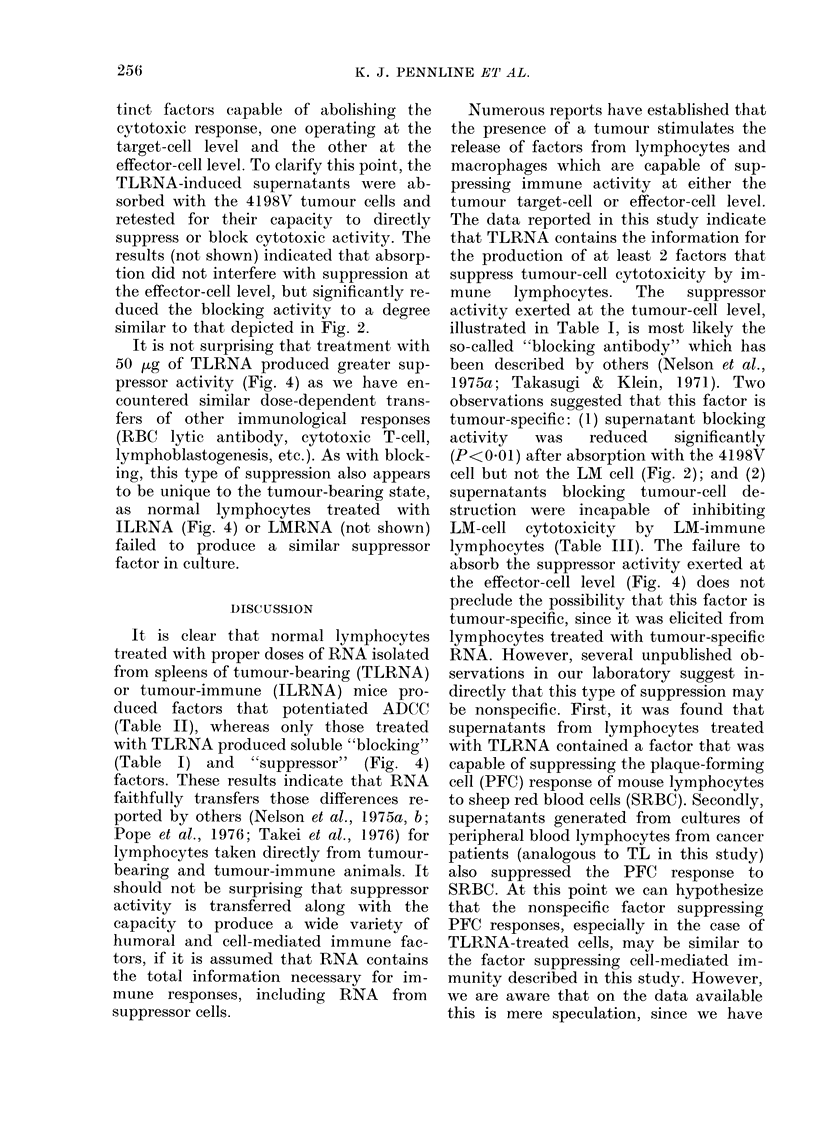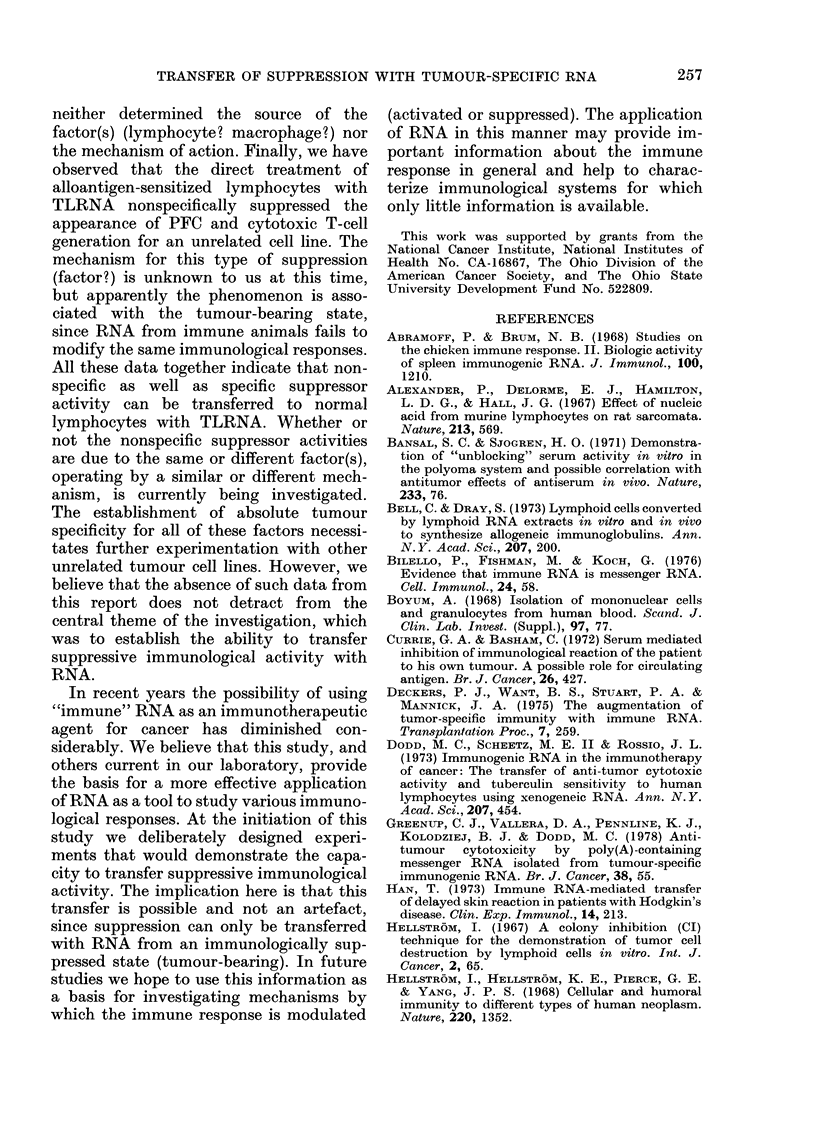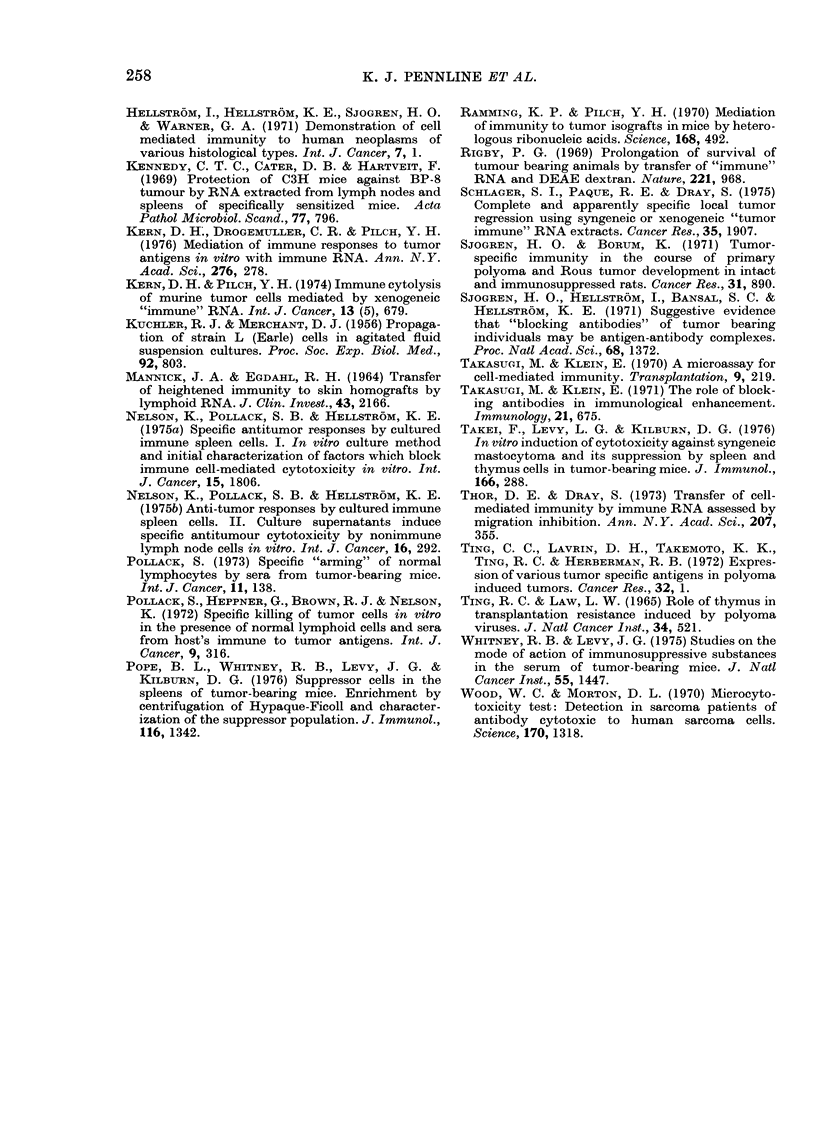# Release of soluble "blocking" and "suppressor" factors from normal lymphocytes treated with RNA from spleens of tumour-bearing mice.

**DOI:** 10.1038/bjc.1979.48

**Published:** 1979-03

**Authors:** K. J. Pennline, S. B. Evans, J. F. Nawrocki, J. C. Rees, C. S. Johnson, D. A. Vallera, M. C. Dodd

## Abstract

**Images:**


*Br. J. Cancer* (1979), **39**, 247

### RELEASE OF SOLUBLE "BLOCKING" AND "SUPPRESSOR" FACTORS FROM NORMAL LYMPHOCYTES TREATED WITH RNA FROM SPLEENS OF TUMOUR-BEARING MICE

K. J. PENNLINE[1], S. B. EVANS[2], J. F. NAWROCKI[3], J. C. REES[4], C. S. JOHNSON[5], D. A. VALLERA[6] AND M. C. DODD

*From the Department of Microbiology, The Ohio State University, Columbus, Ohio* 43210

Received 17 July 1978 Accepted 6 December 1978

**Summary.—RNA extracted from the spleens of tumour-bearing (TLRNA) and tumour-immune (ILRNA) mice was shown to transfer to normal lymphocytes (NL) the ability to produce factors that blocked specific tumour-cell cytotoxicity and mediated specific antibody-dependent cell cytotoxicity (ADCC). Aliquots of normal C3H mouse lymphocytes were treated with TLRNA or ILRNA and cultured *in vitro* in the absence of tumour antigen. Supernatants were collected at 24h intervals and tested in a microcytotoxicity assay for blocking and ADCC activities. Factors that inhibited tumour destruction by specifically sensitized lymphocytes at the level of both the tumour cells and effector cells were demonstrable in culture supernatants of NL pretreated with TLRNA (50 or 100 $\mu$g/4$\times 10^6$ cells) but not ILRNA. However, treatment of NL with either RNA resulted in the production of factors that mediated tumour-specific ADCC. Cytotoxicity testing and absorption studies of the tumour cell and a control cell (LM) indicated that factors mediating ADCC and blocking at the target-cell level were specific for the tumour. Suppressor activity at the effector-cell level was not absorbed by tumour cells and represents a separate and distinct mechanism of immunosuppression. These data indicate that RNA faithfully transfers "suppressive" as well as "positive" types of immune responses that have been reported previously for lymphocytes obtained directly from tumour-bearing and tumour-immune animals.**

OUR LABORATORY and others have demonstrated the ability to transfer humoral and cell-mediated immune responses to normal lymphocytes *in vitro* and *in vivo* by treatment with RNA extracted from the lymphoid tissue of immunized animals. Documented RNA-mediated transfers have been described in various antigen systems such as sheep red blood cells (Abramoff & Brum, 1968; Bell & Dray, 1973) tuberculin (Dodd *et al.*, 1973; Thor & Dray, 1973) allogenic tissue (Mannick & Egdahl, 1964) and tumours (Alexander *et al.*, 1967; Dodd *et al.*, 1973; Kern *et al.*, 1976; Kern & Pilch, 1974; Thor & Dray, 1973). These earlier investigations were based entirely on the transfer of "positive" types of immune responses, in which lytic antibody (Abramoff & Brum, 1968; Bell & Dray, 1973) skin-test reaction (Han, 1973) production of migration-inhibitory factor (Dodd *et al.*,

Present addresses:
[1] Department of Microbiology, Schools of Medicine and Dentistry, Georgetown University, Washington, D.C. 20007.
[2] School of Osteopathic Medicine, Ohio University, Athens, Ohio 45701.
[3] Department of Microbiology, University of Michigan, Ann Arbor, Michigan 48104.
[4] Department of Biology, Catholic University, Washington, D.C. 20011.
[5] Michigan Cancer Foundation, Detroit, Michigan 48201.
[6] Department of Surgery, University of Minnesota, Minneapolis, Minnesota 55455.
Address for correspondence: Dr Matthew C. Dodd, The Department of Microbiology, The Ohio State University, 484 W 12th Avenue, Columbus, Ohio 43210.

1973; Kern *et al.*, 1976; Thor & Dray, 1973) lymphoblastogenesis (Deckers *et al.*, 1975; Dodd *et al.*, 1973) cytotoxic T-cells (Alexander *et al.*, 1967; Dodd *et al.*, 1973; Kern & Pilch, 1974) and protection against tumours (Alexander *et al.*, 1967; Kennedy *et al.*, 1969; Ramming & Pilch, 1970; Rigby, 1969; Schlager *et al.*, 1975) were demonstrated. Credence for these studies is dependent upon the demonstration of antigenic specificity for the transferred response. In a recent report from our laboratory, Greenup *et al.* (1978) using multiple criss-cross experiments with RNA directed at a variety of normal and tumour cell lines, demonstrated that the RNA-transferred cytotoxic response was specific for the eliciting antigen. Similar specificity studies have been made by investigators for many other "positive" types of responses transferred with RNA.

Since suppression of the immune response by soluble factors has become an important and provocative area of research, we began to investigate the possibility of transferring "negative" or "suppressive" types of immune activity with RNA. An animal tumour model was a logical choice in which to initiate such studies, since "positive" and "suppressive" immune responses have been reported. Investigators have demonstrated similar "positive" immunological responses in tumour-immune and tumour-bearing animals such as complement (C′)-dependent cytolytic antibody (Bansal & Sjogren, 1971; Hellstrom *et al.*, 1968; Wood & Morton, 1970) C′-independent lymphocyte-dependent cytotoxic antibody (ADCC) (Pollack, 1973; Pollack *et al.*, 1972) and cytotoxic T-cells (Hellström, 1967; Hellström *et al.*, 1971; Sjogren & Borum, 1971). However, the response in the tumour-bearing host also includes "blocking" or "enhancing" factors, variously described as antibody (Takasugi & Klein, 1971) antigen (Currie & Basham, 1972) or soluble complex of both (Sjogren *et al.*, 1971) and "suppressor" factors released by suppressor cells which effectively abolish the action of specifically sensitized lymphocytes at the level of either the target cell or the effector cell. Production of the former (Nelson *et al.*, 1975*a*) and the latter (Pope *et al.*, 1976; Takei *et al.*, 1976) have been demonstrated *in vitro* by culturing splenic lymphocytes from tumour-bearing animals. In a comparative study, Nelson *et al.* (1975*a*, 1975*b*) showed that while lymphocytes from tumour-immune (IL) and tumour-bearing (TL) mice could produce ADCC antibody in culture, only TL could produce "blocking" factors. Pope *et al.* (1976) and Takei *et al.* (1976) found that suppressor cells isolated from the spleens of tumour-bearing animals elaborated factors that suppressed specific and nonspecific immunological responses at the level of the lymphocyte. Since the spleen was the source of "blocking" and "suppressor" activities in these studies, and the spleen serves as a source of RNA in our studies, we assumed that the information necessary to transfer "suppressive" responses was contained in our RNA preparations. The transfer of such distinctive suppressor activities would provide additional evidence for the hypothesis that "immune" RNA acts as an informational molecule as others have suggested (Bilello *et al.*, 1976; Dodd *et al.*, 1973; Greenup *et al.*, 1978).

The results from this study indicated that normal lymphocytes treated with RNA isolated from spleens of tumour-bearing mice (TLRNA) released soluble factors that "suppressed" cell-mediated immunity at the effector-cell and target-cell levels. The phenomenon was found to be unique to TLRNA and not an artefact, since RNA from spleens of immune animals transferred only "positive" immunological responsiveness.

### MATERIALS AND METHODS

*Animals.*—Inbred male C3H/HeJ mice, 6–8 weeks old, were obtained from Jackson Laboratories, Bar Harbor, Maine and used in all studies.

*Cell lines.*—All the cell lines used in this investigation were of C3H mouse origin.

They included the 4198, the 4198V and the LM cells. The 4198 tumour cell originated from the transformation of C3H cells with an LID strain of the polyoma virus (Ting & Law, 1965). These cells give rise to tumours in C3H mice by 14 days after i.m. injection of $2{\cdot}5 \times 10^4$ cells. The tumour, a fibrosarcoma, has been shown to be free of demonstrable virus by haemagglutination inhibition and plaque-formation assays (Ting & Law, 1965). The 4198V cell, a variant of 4198, arose during *in vitro* passage (Ting *et al.*, 1972). The tumour-associated antigen of 4198V, determined by isotopic antiglobulin absorption, was $8{\cdot}8 \times$ that in the 4198 cells (Ting *et al.*, 1972) making it well suited for immunization and *in vitro* cytotoxicity measurements. The LM cell was cloned from L-cells by Kuchler & Merchant (1956). This syngeneic, non-tumorigenic cell is capable of eliciting an immune response in C3H mice and served as a control in this study.

All cells were maintained as monolayer cultures in RPMI-1640 containing 10% heat-inactivated foetal calf serum (56°C for 30 min) and 2 mM L-glutamine (Grand Island Biological Company, Grand Island, New York).

*Sources of RNA and lymphocytes.*—RNA and lymphocytes were obtained from tumour-bearing or 4198V-immunized and LM-immunized animals. Tumours were induced by i.m. injection of $5 \times 10^4$ 4198 cells, suspended in serum-free RPM1-1640, into one hind leg of C3H mice. Palpable tumours appeared in all mice within 10–12 days. Spleens were harvested from mice 5–7 days after tumour appearance. If lymphocytic RNA was desired, the spleens were immediately frozen in a dry-ice/acetone bath and stored at −70°C until RNA extraction. Lymphocytes used directly for culturing were teased free of the splenic capsule in RPM1-1640, separated from red cells and granulocytes using Ficoll–Hypaque according to Boyum (1968), washed and resuspended to desired concentrations.

The methods for immunizing C3H mice against the 4198V tumour cell and the LM cell were as described in a previous paper (Greenup *et al.*, 1978). RNA and lymphocytes were harvested from the spleens of immune animals as described above. Lymphocytes from 4198V-immune mice were also used as effector cells in all cytotoxicity assays.

Control RNA and lymphocytes were obtained from normal untreated animals.

*RNA extraction.*—RNA was extracted from the frozen mouse spleens (10–12 per extraction) using a modified biphasic extraction in hot phenol as described in a previous paper (Dodd *et al.*, 1973). We have shown that the extracted RNA exhibits a characteristic 3-peak profile (5S, 18S and 28S) on sucrose-density gradients (Dodd *et al.*, 1973; Greenup *et al.*, 1978). The low-molecular-weight (4–6S) RNA contains transfer RNA, the second peak (12–20S) comprises mRNA and smaller ribosomal RNA, and the larger-molecular-weight (20–35S) RNA accounts for larger ribosomal RNA. Previous investigations using fractionation studies have demonstrated that the immunologically active components of the total cellular RNA were confined to the 10–16S sedimentation range (Bilello *et al.*, 1976; Dodd *et al.*, 1973; Kern *et al.*, 1976). In this study normal lymphocytes were treated with specific amounts of whole unfractionated RNA.

*RNA treatment of lymphocytes.*—RNA extracted from the splenic lymphocytes of normal (NLRNA), tumour-bearing (TLRNA), tumour-immune (ILRNA) and LM-immunized (LMRNA) mice was used to treat normal C3H mouse lymphocytes (NL). The procedure for RNA treatment has been described in detail in a previous paper (Greenup *et al.*, 1978). After treatment, the cells were washed and resuspended to a concentration of $5\text{–}8 \times 10^6$ lymphocytes/ml in RPMI-1640 supplemented with 20% heat-inactivated foetal calf serum, 2 mM L-glutamine, 25 mM HEPES (Sigma Chemical Company, St Louis, Missouri) and 100 μg/ml gentamicin (Schering Corporation, Kenilworth, N.J.).

*Lymphocyte culturing.*—A system free of specific antigen was developed in order to detect factors released by RNA-treated lymphocytes maintained in continuous culture. The procedure was a modification of the method used by Nelson *et al.* (1975*a*). Suspensions of RNA-treated lymphocytes ($5\text{–}8 \times 10^6$/ml) were added to 25ml Erlenmeyer flasks in 5ml volumes and incubated in the absence of antigen at 37°C in a humidified atmosphere containing 5% $CO_2$. Supernatants were collected at 24h intervals by transferring the cell suspensions to sterile tubes and centrifuging at 250 *g* for 6 min. The supernatants were removed, filtered, heat-inactivated and stored at −20°C. The cell pellets were resuspended in fresh culture medium and reincubated. Lymphocytes from spleens of normal (NL), tumour-bearing (TL), tumour-immune (IL) and LM-immunized (ILM) mice were maintained in the same manner. Viability of lymphocytes was assessed at each interval of supernatant collection.

*Microcytotoxicity assay.*—A modification of the Takasugi & Klein (1970) microcytotoxicity assay developed in our laboratory has been described in detail by Greenup *et al.* (1978). Briefly, 4198V tumour cells were seeded into wells (100/well) of 3034 microcytotoxicity plates (Falcon Plastics, Cherry Hill, N.J.). The tumour cells were allowed to attach for 12 h, after which specifically immune lymphocytes were added to the wells at an effector cell:target cell ratio of 100:1. After an additional 40 h of incubation the remaining tumour cells were washed, fixed with acetone–alcohol and stained with crystal violet. The cells in the wells of each test row were counted and averaged. The percent cytotoxicity (%C) was calculated as follows:

$$\%C = \frac{\text{(Mean number of tumour cells left in control wells)} - \text{(Mean number of tumour cells left in test wells)}}{\text{(Mean number of tumour cells left in control wells)}} \times 100$$

*Blocking assay.*—Supernatants from the various lymphocyte cultures were tested for their capacity to block the cytotoxic action of specifically tumour-sensitized lymphocytes. Microcytotoxicity plates were seeded with tumour cells as described above. Before the addition of effector cells, 0·01 ml of the various supernatants was added to the appropriate wells and remained in contact with the tumour cells for 30 min. The supernatants were decanted and cytotoxic lymphocytes added. After 40 h of incubation the tests were terminated and the cytotoxicity determined as described previously. The percent blocking (%B) was calculated as follows:

$$\%B = \frac{\text{(\%C in the control supernatants)} - \text{(\%C in the test supernatants)}}{\text{(\%C in the control supernatants)}} \times 100$$

*ADCC assay.*—The ability of the culture supernatants to induce tumour-cell cytotoxicity by normal lympohcytes was determined. The experimental protocol was the same as that described for blocking, with the exception of using normal, nonsensitized lymphocytes as effector cells. The % ADCC was calculated as follows:

$$\%ADCC = \frac{\text{(Mean number of tumour cells in wells with control supernatants)} - \text{(Mean number of tumour cells in wells with test supernatants)}}{\text{(Mean number of tumour cells in wells with control supernatants)}} \times 100$$

*Specificity of RNA-induced supernatant activity.*—Tumour specificity of the elaborated factors was determined in two ways. First, criss-cross experiments were done for all of the above assays, and used the control LM cell (as a target cell) and supernatants from LM-immune lymphocytes and normal lymphocytes treated with LMRNA. Secondly, absorption studies were made by incubating 2 ml of the supernatants from cells treated with tumour-specific ILRNA or TLRNA with $10^7$ tumour cells or LM cells for 45 min at 37°C. The supernatants were centrifuged free of cells, filtered and retested for blocking and ADCC activity.

*Treatment of effector cells with supernatants.*—In order to determine the effect of supernatants on the cell-mediated cytotoxic response at the effector-cell level, tumour-immune lymphocytes were incubated for 30 min in 2 ml of the various supernatants. The lymphocytes were then washed ×3 in serum-free medium, resuspended in complete medium ($10^6$/ml) and tested on plated 4198V cells to assess cytotoxic potential as described above.

*Analysis of data.*—The data presented in this study represent the results of two experiments. All experiments were repeated at least 3–4 times in order to establish the reproducibility of the systems. Statistical analysis of the data was by Student's *t* test.

### RESULTS

### *Viability of cultured lymphocytes*

It was realised initially that the viability of RNA-treated cells in an antigen-free culture would be a limiting factor in the time span of the experiments. As is shown

FIG. 1.—Viability of untreated lymphocytes (TL, IL and NL) and normal lymphocytes treated with tumour-specific RNA (TLRNA and ILRNA) at 24h intervals of culture. % viability was determined by trypan-blue dye exclusion/100 cells. Columns represent the mean of 6 counts from 2 experiments. Three repetitions of this experiment yielded similar results.

in Fig. 1, 74–83% of splenic lymphocytes from tumour-bearing (TL) or tumour-immune (IL) mice remained viable for 4 days, whilst less than 50% of normal lymphocytes (NL) survived for 48 h under the same conditions. Fig. 1 also shows that treatment of NL with tumour-specific RNA from immune or tumour-bearing mouse spleens (ILRNA or TLRNA), in 50μg or 100μg quantities, increased the viability from 43% to 68–85% at 48 h, whereas treatment with RNA from normal mouse spleens (NLRNA) had no detectable effect on lymphocyte viability (40% at 48 h). It is possible that RNA from sensitized lymphocytes may provide some of the information responsible for the sustained viability of these cells under these conditions.

*Demonstration of blocking activity in supernatants*

Supernatants from untreated and RNA-treated cells collected at 24, 48, 72 and 96 h of culture were tested simultaneously for the presence of factors capable of abrogating the cytotoxic action of tumour-immune lymphocytes (IL) at the tumour-cell level. Table I summarizes the results of 2 experiments and illustrates the cytotoxicity of supernatant-exposed or unexposed tumour cells by IL. The data indicate that treatment of normal lymphocytes (NL) with RNA extracted from spleens of tumour-bearing mice (TLRNA), in 50μg or 100μg quantities, produced supernatants containing blocking activity, as illustrated by a marked reduction of cytotoxicity by IL (24–72 h supernatants) when compared to control supernatants (from NL treated with NLRNA). The blocking effect was also demonstrable with supernatants from lymphocytes taken directly from tumour-bearing animals (TL) which has been reported by Nelson *et al.* (1975*a*). It is interesting to note that the observed blocking activity produced by TLRNA-treated cells was detectable earlier (24 h compared to 48 h) and present in greater amounts (59·3–60·7% blocking compared to 34·6–41·9%) than that produced by TL, although production by the latter was more sustained, possibly due to their longer survival. The last significant blocking activity produced by TLRNA-treated cells coincides fairly well with the 50% loss of viability (Fig. 1). The generation of blocking activity by TLRNA-treated lymphocytes was found to be a reproducible event throughout this study, and in 4 additional experiments.

Table I also illustrates the lack of blocking activity in supernatants from NL treated with splenic RNA from tumour-immune mice (ILRNA) or from lymphocytes taken directly from these animals (IL). Instances of high "negative blocking activity" (as great as −30·9% by IL at 72 h) could possibly represent increased cytotoxic activity attributed to

TABLE I.—*Blocking of specific tumour-cell cytotoxicity by supernatants from RNA-treated and tumour-sensitized lymphocytes*

| Effector cell | Supernatant from | Tumour cells/well remaining (Mean ± s.e.)[1] | | | | % Cytotoxicity by IL | | | | % Blocking | | | |
|---|---|---|---|---|---|---|---|---|---|---|---|---|---|
| NL | — | 67 ± 6 | | | | Control | | | | | | | |
| IL | — | 37 ± 4 | | | | 44·7 | | | | | | | |
| | | | | | | After exposure to supernatant and IL | | | | | | | |
| | NL treated with: | 24 | 48 | 72 | 96[2] | 24 | 48 | 72 | 96 | 24 | 48 | 72 | 96 |
| | | | | | | | | | | RNA-treated control | | | |
| IL | 100 μg NLRNA | 37 ± 4 | 39 ± 3 | 40 ± 5 | 34 ± 5 | 44·7 | 41·8 | 40·3 | 49·2 | | | | |
| IL | 100 μg ILRNA | 36 ± 5 | 37 ± 4 | 36 ± 4 | 33 ± 3 | 46·3 | 44·7 | 46·3 | 50·7 | −3·6 | −6·9 | −14·8 | −3·0 |
| IL | 50 μg TLRNA | 48 ± 4 | 51 ± 3 | 56 ± 4 | 36 ± 4 | 28·3 | 23·9 | 16·4 | 46·3 | 36·6† | 42·8† | 59·3* | 5·9 |
| IL | 100 μg TLRNA | 50 ± 6 | 56 ± 5 | 42 ± 4 | 35 ± 4 | 25·4 | 16·4 | 37·3 | 47·8 | 43·2† | 60·7* | 7·4 | 2·8 |
| | untreated: | | | | | | | | | Untreated control | | | |
| IL | NL | 39 ± 5 | 38 ± 3 | 38 ± 4 | 36 ± 5 | 41·8 | 43·3 | 43·3 | 46·3 | | | | |
| IL | IL | 40 ± 5 | 35 ± 3 | 29 ± 3 | 33 ± 3 | 40·3 | 47·8 | 56·7 | 50·7 | 3·6 | −9·2 | −30·9 | −9·5 |
| IL | TL | 40 ± 5 | 48 ± 5 | 47 ± 4 | 49 ± 3 | 40·3 | 28·3 | 29·8 | 26·9 | 3·6 | 34·6† | 31·2† | 41·9† |

[1] Mean of 20 replicate wells.
[2] Hour of culture that supernatants were collected.
* $P < 0{\cdot}01$.
† $P < 0{\cdot}05$,

TABLE II.—*ADCC activity associated with supernatants from RNA-treated and tumour-sensitized lymphocytes*

| Effector cell | Supernatant from | Tumour cells/well remaining (Mean ± s.e.)[1] | | | | % ADCC | | | |
|---|---|---|---|---|---|---|---|---|---|
| NL | none | 57 ± 5 | | | | | | | |
| | | After exposure to supernatant and NL | | | | | | | |
| | NL treated with: | 24 | 48 | 72 | 96[2] | 24 | 48 | 72 | 96 |
| NL | 100 μg NLRNA | 53 ± 4 | 59 ± 4 | 57 ± 3 | nd[3] | RNA-treated control | | | |
| NL | 100 μg ILRNA | 35 ± 3 | 35 ± 4 | 49 ± 3 | nd | 33·9* | 40·7* | 14·0 | — |
| NL | 100 μg TLRNA | 31 ± 3 | 43 ± 4 | 51 ± 5 | nd | 41·5* | 27·1† | 10·5 | — |
| | Untreated: | | | | | | | | |
| NL | NL | 55 ± 5 | 55 ± 4 | 53 ± 4 | 58 ± 4 | Untreated control | | | |
| NL | IL | 48 ± 4 | 34 ± 3 | 28 ± 2 | 34 ± 3 | 12·7 | 38·2* | 47·2* | 41·4* |
| NL | TL | 50 ± 5 | 40 ± 3 | 30 ± 2 | 31 ± 2 | 9·0 | 27·3† | 43·4* | 46·5* |

1 Mean of 20 replicate wells.
2 Hour of culture that supernatants were collected.
3 Not done.
* $P < 0·01$.
† $P < 0·05$.

TABLE III.—*Tumour-cell specificity of supernatant factors mediating blocking and ADCC*

| | Target cells | Supernatant from | Target cells/well[1] remaining after exposure to supernatant and: (Mean ± s.e.) NL | IL | % C[2] by IL | % B[6] | % ADCC |
|---|---|---|---|---|---|---|---|
| A | | NL Treated with: | | | | | |
| | 4198V | 100 μg NLRNA | **66 ± 4**[4] | 39 ± 3 | **40·9** | | |
| | | 100 μg ILRNA | 46 ± 4 | 34 ± 3 | 48·4 | −18·3 | 30·3* |
| | | 50 μg TLRNA | nd[5] | 55 ± 4 | 16·6 | 59·4* | nd |
| | | 100 μg TLRNA | 47 ± 3 | 53 ± 4 | 19·7 | 51·8* | 28·8* |
| | | 100 μg LMRNA | 69 ± 4 | 37 ± 3 | 43·9 | −7·3 | −4·5 |
| | | Untreated: | | | | | |
| | | NL | **68 ± 5** | 38 ± 3 | **44·1** | | |
| | | IL | 42 ± 4 | 28 ± 2 | 58·8 | −33·3 | 38·2* |
| | | TL | 45 ± 4 | 51 ± 4 | 25·0 | 43·3* | 33·8* |
| | | ILM | 66 ± 5 | 35 ± 3 | 48·5 | −9·8 | 2·9 |
| | | | NL | ILM | by ILM | | |
| B | | NL Treated with: | | | | | |
| | LM | 100 μg NLRNA | **82 ± 6** | 50 ± 5 | **39·0** | | |
| | | 100 μg ILRNA | 79 ± 4 | 52 ± 4 | 36·6 | 6·1 | 3·6 |
| | | 50 μg TLRNA | nd | 53 ± 4 | 35·4 | 9·2 | nd |
| | | 100 μg TLRNA | 81 ± 5 | 52 ± 4 | 36·6 | 6·1 | 1·2 |
| | | 100 μg LMRNA | 57 ± 4 | 45 ± 3 | 40·2 | −15·6 | 30·5* |
| | | Untreated: | | | | | |
| | | NL | **82 ± 5** | 50 ± 5 | **40·2** | | |
| | | IL | 80 ± 4 | 49 ± 3 | 41·5 | −3·2 | 2·4 |
| | | TL | 84 ± 4 | 51 ± 4 | 37·8 | 5·9 | −2·4 |
| | | ILM | 54 ± 4 | 47 ± 3 | 42·7 | −6·2 | 34·1* |

1 Mean of 20 replicate wells.
2 % C—Cytotoxicity.
3 % B—Blocking.
4 Means in bold type serve as respective controls.
5 Not done.
* $P < 0·01$.

factors that potentiated cytotoxicity by resident normal effector cells (ADCC, Table II) present in the immune population.

*Demonstration of ADCC activity in supernatants*

Supernatants described in Table I were tested for ADCC activity. The data from 2 typical experiments summarized in Table II illustrate that non-immune lymphoid cells (NL) displayed significant cytotoxicity for tumour cells that had been previously exposed to supernatants from NL treated with 100 μg of ILRNA (33·9–40·7% ADCC) and TLRNA (27·1–41·5% ADCC) when compared to control supernatants (from NL treated with NLRNA). As reported by Nelson *et al.* (1975*b*) ADCC activity was also present in supernatants of IL and TL. Peak activity for RNA-treated lymphocytes (40·7–41·5%) was confined to the 24 h and 48 h periods, whilst supernatants from IL and TL exhibited greatest ADCC activity at 72 h (47·2%) and 96 h (46·5%) respectively. As with blocking activity, the rapid decrease in production by RNA-treated lymphocytes may be associated with the decreased viability of these cells (Fig. 1).

*Specificity of factors that block cell-mediated immunity and potentiate ADCC*

Fresh 48h supernatants were generated from normal lymphocytes treated with TLRNA and ILRNA as well as from TL and IL. In addition, supernatants were generated from normal lymphocytes treated with splenic RNA from LM-cell-immunized mice (LMRNA) and from lymphocytes obtained directly from these animals (ILM). All supernatants were tested in a "criss-cross" pattern against the 4198V and LM target cells. As can be seen in Table III, supernatants arising from RNA-treated lymphocytes exhibiting blocking and ADCC activity for the 4198V tumour cell (Table III, A) did not exert either effect on the LM target cell (Table III, B). Likewise, supernatants derived from LMRNA-treated cells dis-

FIG. 2.—Blocking of specific 4198V tumour-cell cytotoxicity by supernatants from normal lymphocytes (NL) treated with 50 and 100 μg of TLRNA before and after absorption with 4198V or LM cells. % blocking was calculated as described in the text, with supernatants from NL treated with NLRNA as the control. Columns represent the mean of 20 replicate wells from 2 experiments and the bars represent the range. Probability that the difference between unabsorbed and 4198V-absorbed groups were due to chance was $<0\cdot05$. Similar results were obtained from 3 additional experiments.

FIG. 3.—ADCC activity for the 4198V tumour cells associated with supernatants from normal lymphocytes (NL) treated with 100 μg of TLRNA or ILRNA before and after absorption with 4198V and LM cells. % ADCC was calculated as described in the text, with supernatants from NL treated with NLRNA as the control. Columns represent the mean of 20 replicate wells from 2 experiments, and the bars represent the range. Probability that the differences between unabsorbed and 4198V-absorbed groups were due to chance was $<0\cdot01$. Similar results were obtained from 3 additional experiments.

playing ADCC activity for the LM cell (Table III, B) failed to induce cytotoxicity of the 4198V cells by the same mechanism (Table III, A). Supernatants from TL, IL and ILM exhibited a similar trend when cross-tested. It is interesting to note that no significant blocking activity was associated with the supernatants generated against the LM cell. As this cell is not tumorigenic, the absence of blocking activity provides additional evidence for the distinction between the tumour-bearing and immune states described in a similar fashion by Nelson *et al.* (1975*a*, *b*).

To illustrate further the specificity of the elaborated factors, aliquots of supernatants from RNA-treated cells shown in Table III were absorbed with $10^7$ LM or 4198V cells and retested for blocking and ADCC activities. Results illustrated in Fig. 2 show that blocking activity produced by lymphocytes treated with 50μg or 100μg of TLRNA was reduced significantly (59·4% to 25·2% and 51·8% to 8·2% respectively) only when the supernatants were absorbed with tumour cells and not LM cells. Likewise, the LM cell did not remove tumour-specific ADCC activity. However, absorption with the 4198V cell reduced this activity from 28·8% to 4·4% and from 30·3% to 2·8% in supernatants from TLRNA- and ILRNA-treated cells respectively (Fig. 3). In similar experiments, ADCC activity for the LM cell was not removed by absorption with the 4198V cell but was completely abolished after absorption with the LM cell (data not shown).

*Suppression of cytotoxicity at the effector-cell level by supernatant fluids*

In previous experiments, TLRNA-induced blocking activity was assessed at the target-cell level by pretreating the target cells with supernatants before the addition of cytotoxic lymphocytes. In the light of observations by Pope *et al.* (1976) and Takei *et al.* (1976) who demonstrated that immune responses could be suppressed by soluble factors at the effector-cell level, we designed experiments that

FIG. 4.—Cytotoxic activity of 4198V-immune lymphocytes after exposure to culture supernatants from normal lymphocytes (NL) treated with 50 (▩) or 100 μg (▨) of TLRNA or 100 μg of ILRNA (■). % suppression was calculated in the same fashion as blocking with control supernatants from NL treated with NLRNA. Columns represent the mean of 20 replicate wells from 2 experiments and the bars represent the range. Probability that the differences between the control and test supernatants were due to chance was <0·001 (50 μg TLRNA) and $P<0·05$ (100 μg TLRNA). Similar results were obtained from 4 additional experiments.

would determine whether TLRNA contained the information necessary to allow normal lymphocytes to produce factors capable of suppressing the cytotoxic response of tumour-immune lymphocytes (IL). IL were incubated for 30 min with fresh 48h supernatants from RNA-treated cells, washed and then tested on plated 4198V target cells. As can be seen in Fig. 4, the cytotoxic activity of IL was suppressed significantly (80%, $P<0·001$) after exposure to supernatants from cells treated with 50 μg of TLRNA, and was ~20% higher than inhibition seen in Table III when blocking (~60%) was assessed at the target-cell level. Direct suppression of cytotoxicity was also apparent after IL exposure to supernatants of cells treated with 100 μg of TLRNA (27%, $P<0·05$) but, in contrast, was considerably lower than blocking activity shown in Table III (51·8%) with the same dose of RNA. Taken together these data suggest the presence of 2 distinct factors capable of abolishing the cytotoxic response, one operating at the target-cell level and the other at the effector-cell level. To clarify this point, the TLRNA-induced supernatants were absorbed with the 4198V tumour cells and retested for their capacity to directly suppress or block cytotoxic activity. The results (not shown) indicated that absorption did not interfere with suppression at the effector-cell level, but significantly reduced the blocking activity to a degree similar to that depicted in Fig. 2.

It is not surprising that treatment with 50 $\mu$g of TLRNA produced greater suppressor activity (Fig. 4) as we have encountered similar dose-dependent transfers of other immunological responses (RBC lytic antibody, cytotoxic T-cell, lymphoblastogenesis, etc.). As with blocking, this type of suppression also appears to be unique to the tumour-bearing state, as normal lymphocytes treated with ILRNA (Fig. 4) or LMRNA (not shown) failed to produce a similar suppressor factor in culture.

### DISCUSSION

It is clear that normal lymphocytes treated with proper doses of RNA isolated from spleens of tumour-bearing (TLRNA) or tumour-immune (ILRNA) mice produced factors that potentiated ADCC (Table II), whereas only those treated with TLRNA produced soluble "blocking" (Table I) and "suppressor" (Fig. 4) factors. These results indicate that RNA faithfully transfers those differences reported by others (Nelson *et al.*, 1975*a*, *b*; Pope *et al.*, 1976; Takei *et al.*, 1976) for lymphocytes taken directly from tumour-bearing and tumour-immune animals. It should not be surprising that suppressor activity is transferred along with the capacity to produce a wide variety of humoral and cell-mediated immune factors, if it is assumed that RNA contains the total information necessary for immune responses, including RNA from suppressor cells.

Numerous reports have established that the presence of a tumour stimulates the release of factors from lymphocytes and macrophages which are capable of suppressing immune activity at either the tumour target-cell or effector-cell level. The data reported in this study indicate that TLRNA contains the information for the production of at least 2 factors that suppress tumour-cell cytotoxicity by immune lymphocytes. The suppressor activity exerted at the tumour-cell level, illustrated in Table I, is most likely the so-called "blocking antibody" which has been described by others (Nelson *et al.*, 1975*a*; Takasugi & Klein, 1971). Two observations suggested that this factor is tumour-specific: (1) supernatant blocking activity was reduced significantly ($P<0{\cdot}01$) after absorption with the 4198V cell but not the LM cell (Fig. 2); and (2) supernatants blocking tumour-cell destruction were incapable of inhibiting LM-cell cytotoxicity by LM-immune lymphocytes (Table III). The failure to absorb the suppressor activity exerted at the effector-cell level (Fig. 4) does not preclude the possibility that this factor is tumour-specific, since it was elicited from lymphocytes treated with tumour-specific RNA. However, several unpublished observations in our laboratory suggest indirectly that this type of suppression may be nonspecific. First, it was found that supernatants from lymphocytes treated with TLRNA contained a factor that was capable of suppressing the plaque-forming cell (PFC) response of mouse lymphocytes to sheep red blood cells (SRBC). Secondly, supernatants generated from cultures of peripheral blood lymphocytes from cancer patients (analogous to TL in this study) also suppressed the PFC response to SRBC. At this point we can hypothesize that the nonspecific factor suppressing PFC responses, especially in the case of TLRNA-treated cells, may be similar to the factor suppressing cell-mediated immunity described in this study. However, we are aware that on the data available this is mere speculation, since we have neither determined the source of the factor(s) (lymphocyte? macrophage?) nor the mechanism of action. Finally, we have observed that the direct treatment of alloantigen-sensitized lymphocytes with TLRNA nonspecifically suppressed the appearance of PFC and cytotoxic T-cell generation for an unrelated cell line. The mechanism for this type of suppression (factor?) is unknown to us at this time, but apparently the phenomenon is associated with the tumour-bearing state, since RNA from immune animals fails to modify the same immunological responses. All these data together indicate that nonspecific as well as specific suppressor activity can be transferred to normal lymphocytes with TLRNA. Whether or not the nonspecific suppressor activities are due to the same or different factor(s), operating by a similar or different mechanism, is currently being investigated. The establishment of absolute tumour specificity for all of these factors necessitates further experimentation with other unrelated tumour cell lines. However, we believe that the absence of such data from this report does not detract from the central theme of the investigation, which was to establish the ability to transfer suppressive immunological activity with RNA.

In recent years the possibility of using "immune" RNA as an immunotherapeutic agent for cancer has diminished considerably. We believe that this study, and others current in our laboratory, provide the basis for a more effective application of RNA as a tool to study various immunological responses. At the initiation of this study we deliberately designed experiments that would demonstrate the capacity to transfer suppressive immunological activity. The implication here is that this transfer is possible and not an artefact, since suppression can only be transferred with RNA from an immunologically suppressed state (tumour-bearing). In future studies we hope to use this information as a basis for investigating mechanisms by which the immune response is modulated (activated or suppressed). The application of RNA in this manner may provide important information about the immune response in general and help to characterize immunological systems for which only little information is available.

This work was supported by grants from the National Cancer Institute, National Institutes of Health No. CA-16867, The Ohio Division of the American Cancer Society, and The Ohio State University Development Fund No. 522809.

### REFERENCES

ABRAMOFF, P. & BRUM, N. B. (1968) Studies on the chicken immune response. II. Biologic activity of spleen immunogenic RNA. *J. Immunol.*, **100**, 1210.

ALEXANDER, P., DELORME, E. J., HAMILTON, L. D. G., & HALL, J. G. (1967) Effect of nucleic acid from murine lymphocytes on rat sarcomata. *Nature*, **213**, 569.

BANSAL, S. C. & SJOGREN, H. O. (1971) Demonstration of "unblocking" serum activity *in vitro* in the polyoma system and possible correlation with antitumor effects of antiserum *in vivo*. *Nature*, **233**, 76.

BELL, C. & DRAY, S. (1973) Lymphoid cells converted by lymphoid RNA extracts *in vitro* and *in vivo* to synthesize allogeneic immunoglobulins. *Ann. N.Y. Acad. Sci.*, **207**, 200.

BILELLO, P., FISHMAN, M. & KOCH, G. (1976) Evidence that immune RNA is messenger RNA. *Cell. Immunol.*, **24**, 58.

BOYUM, A. (1968) Isolation of mononuclear cells and granulocytes from human blood. *Scand. J. Clin. Lab. Invest.* (Suppl.), **97**, 77.

CURRIE, G. A. & BASHAM, C. (1972) Serum mediated inhibition of immunological reaction of the patient to his own tumour. A possible role for circulating antigen. *Br. J. Cancer*, **26**, 427.

DECKERS, P. J., WANT, B. S., STUART, P. A. & MANNICK, J. A. (1975) The augmentation of tumor-specific immunity with immune RNA. *Transplantation Proc.*, **7**, 259.

DODD, M. C., SCHEETZ, M. E. II & ROSSIO, J. L. (1973) Immunogenic RNA in the immunotherapy of cancer: The transfer of anti-tumor cytotoxic activity and tuberculin sensitivity to human lymphocytes using xenogeneic RNA. *Ann. N.Y. Acad. Sci.*, **207**, 454.

GREENUP, C. J., VALLERA, D. A., PENNLINE, K. J., KOLODZIEJ, B. J. & DODD, M. C. (1978) Anti-tumour cytotoxicity by poly(A)-containing messenger RNA isolated from tumour-specific immunogenic RNA. *Br. J. Cancer*, **38**, 55.

HAN, T. (1973) Immune RNA-mediated transfer of delayed skin reaction in patients with Hodgkin's disease. *Clin. Exp. Immunol.*, **14**, 213.

HELLSTRÖM, I. (1967) A colony inhibition (CI) technique for the demonstration of tumor cell destruction by lymphoid cells *in vitro*. *Int. J. Cancer*, **2**, 65.

HELLSTRÖM, I., HELLSTRÖM, K. E., PIERCE, G. E. & YANG, J. P. S. (1968) Cellular and humoral immunity to different types of human neoplasm. *Nature*, **220**, 1352.

HELLSTRÖM, I., HELLSTRÖM, K. E., SJOGREN, H. O. & WARNER, G. A. (1971) Demonstration of cell mediated immunity to human neoplasms of various histological types. *Int. J. Cancer*, **7**, 1.

KENNEDY, C. T. C., CATER, D. B. & HARTVEIT, F. (1969) Protection of C3H mice against BP-8 tumour by RNA extracted from lymph nodes and spleens of specifically sensitized mice. *Acta Pathol Microbiol. Scand.*, **77**, 796.

KERN, D. H., DROGEMULLER, C. R. & PILCH, Y. H. (1976) Mediation of immune responses to tumor antigens *in vitro* with immune RNA. *Ann. N.Y. Acad. Sci.*, **276**, 278.

KERN, D. H. & PILCH, Y. H. (1974) Immune cytolysis of murine tumor cells mediated by xenogeneic "immune" RNA. *Int. J. Cancer*, **13** (5), 679.

KUCHLER, R. J. & MERCHANT, D. J. (1956) Propagation of strain L (Earle) cells in agitated fluid suspension cultures. *Proc. Soc. Exp. Biol. Med.*, **92**, 803.

MANNICK, J. A. & EGDAHL, R. H. (1964) Transfer of heightened immunity to skin homografts by lymphoid RNA. *J. Clin. Invest.*, **43**, 2166.

NELSON, K., POLLACK, S. B. & HELLSTRÖM, K. E. (1975*a*) Specific antitumor responses by cultured immune spleen cells. I. *In vitro* culture method and initial characterization of factors which block immune cell-mediated cytotoxicity *in vitro*. *Int. J. Cancer*, **15**, 1806.

NELSON, K., POLLACK, S. B. & HELLSTRÖM, K. E. (1975*b*) Anti-tumor responses by cultured immune spleen cells. II. Culture supernatants induce specific antitumour cytotoxicity by nonimmune lymph node cells *in vitro*. *Int. J. Cancer*, **16**, 292.

POLLACK, S. (1973) Specific "arming" of normal lymphocytes by sera from tumor-bearing mice. *Int. J. Cancer*, **11**, 138.

POLLACK, S., HEPPNER, G., BROWN, R. J. & NELSON, K. (1972) Specific killing of tumor cells *in vitro* in the presence of normal lymphoid cells and sera from host's immune to tumor antigens. *Int. J. Cancer*, **9**, 316.

POPE, B. L., WHITNEY, R. B., LEVY, J. G. & KILBURN, D. G. (1976) Suppressor cells in the spleens of tumor-bearing mice. Enrichment by centrifugation of Hypaque-Ficoll and characterization of the suppressor population. *J. Immunol.*, **116**, 1342.

RAMMING, K. P. & PILCH, Y. H. (1970) Mediation of immunity to tumor isografts in mice by heterologous ribonucleic acids. *Science*, **168**, 492.

RIGBY, P. G. (1969) Prolongation of survival of tumour bearing animals by transfer of "immune" RNA and DEAE dextran. *Nature*, **221**, 968.

SCHLAGER, S. I., PAQUE, R. E. & DRAY, S. (1975) Complete and apparently specific local tumor regression using syngeneic or xenogeneic "tumor immune" RNA extracts. *Cancer Res.*, **35**, 1907.

SJOGREN, H. O. & BORUM, K. (1971) Tumor-specific immunity in the course of primary polyoma and Rous tumor development in intact and immunosuppressed rats. *Cancer Res.*, **31**, 890.

SJOGREN, H. O., HELLSTRÖM, I., BANSAL, S. C. & HELLSTRÖM, K. E. (1971) Suggestive evidence that "blocking antibodies" of tumor bearing individuals may be antigen-antibody complexes. *Proc. Natl Acad. Sci.*, **68**, 1372.

TAKASUGI, M. & KLEIN, E. (1970) A microassay for cell-mediated immunity. *Transplantation*, **9**, 219.

TAKASUGI, M. & KLEIN, E. (1971) The role of blocking antibodies in immunological enhancement. *Immunology*, **21**, 675.

TAKEI, F., LEVY, L. G. & KILBURN, D. G. (1976) *In vitro* induction of cytotoxicity against syngeneic mastocytoma and its suppression by spleen and thymus cells in tumor-bearing mice. *J. Immunol.*, **166**, 288.

THOR, D. E. & DRAY, S. (1973) Transfer of cell-mediated immunity by immune RNA assessed by migration inhibition. *Ann. N.Y. Acad. Sci.*, **207**, 355.

TING, C. C., LAVRIN, D. H., TAKEMOTO, K. K., TING, R. C. & HERBERMAN, R. B. (1972) Expression of various tumor specific antigens in polyoma induced tumors. *Cancer Res.*, **32**, 1.

TING, R. C. & LAW, L. W. (1965) Role of thymus in transplantation resistance induced by polyoma viruses. *J. Natl Cancer Inst.*, **34**, 521.

WHITNEY, R. B. & LEVY, J. G. (1975) Studies on the mode of action of immunosuppressive substances in the serum of tumor-bearing mice. *J. Natl Cancer Inst.*, **55**, 1447.

WOOD, W. C. & MORTON, D. L. (1970) Microcytotoxicity test: Detection in sarcoma patients of antibody cytotoxic to human sarcoma cells. *Science*, **170**, 1318.